# Disseminated Coccidioidomycosis Presenting as Septic Shock with Multiorgan Failure

**DOI:** 10.1155/2021/8837493

**Published:** 2021-04-15

**Authors:** Olufemi Aduroja, Jerome Okudo, Angelica Padilla

**Affiliations:** ^1^Department of Nephrology, Texas Tech University Health Sciences Center, 2000B Transmountain Road,Suite 465, El Paso, TX 79911, USA; ^2^School of Public Health, University of Texas, 1200 Pressler Street, Houston, TX 77030, USA; ^3^Department of Pathology, Texas Tech University Health Sciences Center, 4800 Alberta Avenue, El Paso, TX 79905, USA

## Abstract

Coccidioidomycosis is a fungal infection caused by *Coccidioides immitis* and *Coccidioides posadasii.* While infections are usually mild, severe disease occurs in immunocompromised patients. Dissemination is associated with severe morbidity and mortality. Because of the tendency of this disease to imitate many diseases, diagnosis may be difficult on presentation. We present a case of disseminated coccidioidomycosis in a patient who was initially managed as miliary tuberculosis. In endemic areas, coccidioidomycosis is one of the two top differentials for miliary micronodular distribution on chest imaging. The patient was a recently diagnosed HIV positive patient and presented to the hospital with multiorgan failure, septic shock, and acute respiratory distress syndrome. He rapidly deteriorated and died within three days of presentation at the emergency department.

## 1. Introduction

Coccidioidomycosis is well documented to be endemic in California, Southern Arizona and New Mexico, Utah, Nevada, and Texas in the United States [1–5]. It is a fungal infection and infected patients inhale the arthroconidia into the lungs usually in dry weather.

This disease is referred to as the great imitator because of its overlapping symptoms and signs with many other conditions. This disease can infect both immunocompetent and immunocompromised individuals [6–8]. When patients have primary infection, they are usually either asymptomatic, have mild respiratory infections in 60% of the cases, or have clinical presentations for which treatment with continuous antifungal medication is required [1–3, 9, 10]. Disseminated disease is uncommon and make up nearly 5% of recognized infections. Certain characteristics predispose patients to developing disseminated disease and they include increased age, occupational exposure to dust, men of Filipino or African origin, and those with reduced immunity (HIV patients, patients on immunosuppressive medications, transplant recipients, and pregnant patients in their third trimester) [1, 11, 12].

This case is significant because of the following reasons: (1) though coccidioidomycosis has been reported in western Texas, physicians are not required to report it because it is present only in west Texas and not the entire state. (2) El Paso (the city in west Texas in which the patient presented with the disease) has the largest population in this part of Texas. (3) There has neither been a public health prevention intervention nor awareness for the disease in the state on a wider scale [13]. (4) The patient presented in septic shock and in our detailed search of medical literature for coccidioidomycosis cases presenting with septic shock; we could not find many documented cases.

## 2. Case Report

Our patient was a 61-year-old Hispanic man who had not seen a health care provider in many years and presented with one month history of vague abdominal pain, 20 pound weight loss in 1 month, and generalized weakness with malaise as well as occasional cough that worsened and became productive of clear sputum over the last week before presentation. He also reported occasional episodes of nausea and nonbloody diarrhea. He denied any fevers, chills, or rash but reported some arthralgias. The patient was born in Chihuahua, Mexico, but had lived in Texas, USA, since age seven. He was divorced, estranged from his children, and lived alone. There was no significant tobacco or alcohol use but he confirmed exposure to high intensity dust when he used to work in a ceramic and tile factory without wearing a mask for the last five years.

He was evaluated by a private community physician a few days before presentation to our hospital who prescribed for him a combination of oral ciprofloxacin and metronidazole without specific diagnostic testing. The patient did not have any improvement so he presented to the emergency room.

In the emergency department, his temperature was 98.3 F, blood pressure 84/61 mmHg, pulse rate 128/minute, and respiratory rate 27/minute with pulse oximetry 92% on ambient air. He was acutely ill-looking but alert. While lung examination was limited on admission due to COVID-19 restrictions and the use of disposable stethoscopes, there were decreased air sounds but no rales. His abdominal examination was benign but positive findings were significant for large lymph nodes in the cervical, supraclavicular, inguinal, and axillary areas, some measuring more than 3 cm in diameter. Laboratory findings on admission showed white blood cell count was 8,900/mm^3^ with 80% neutrophils, 0.5% band forms, and 11% eosinophils, hemoglobin was 8.9 gm/dL, hematocrit was 27.6%, and platelet count was 447,000/mm^3^. Electrolytes showed bicarbonate of 17, blood urea 54, creatinine 1.5, albumin 2.2, lipase 14, lactic acid 2.5, ALT 57, and AST 139. Procalcitonin was 6.1.

**Figure 1:**
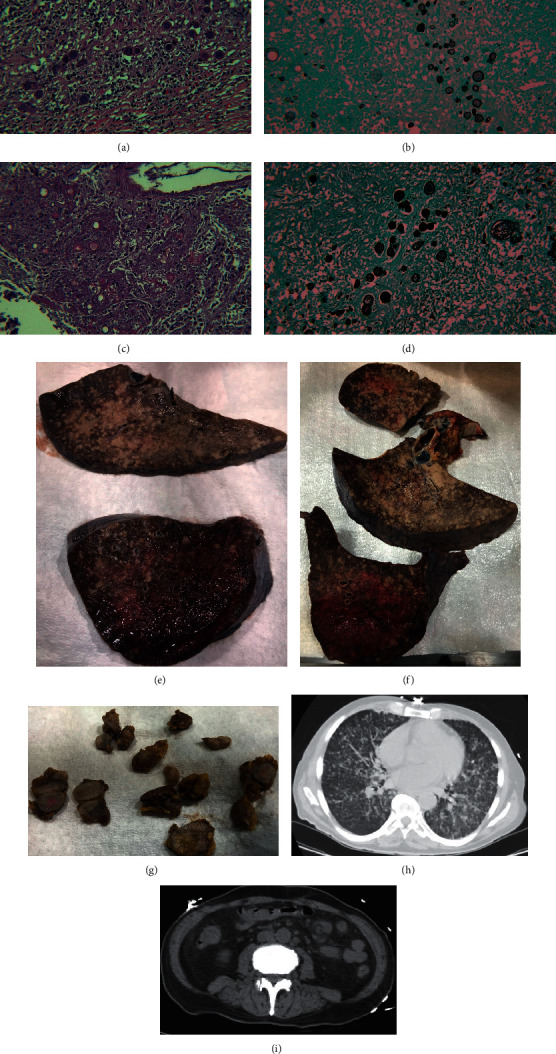
Autopsy findings and figures. (a) The normal lymph node architecture is replaced with granulomatous inflammation with abundant Coccidioides fungal organisms (hematoxylin and eosin stain 20X magnification. (b) Lymph node: Grocott-Gomori's methenamine silver stain (GMS) highlights Coccidioides organisms at 20X magnification. Note empty spherules and spherules filled with endospores. (c) Lung alveolar spaces are filled with caseating granulomatous inflammation with abundant Coccidioides spherules with and without endospores (hematoxylin and eosin stain 20X magnification). (d) Lung: GMS stain highlights Coccidioides spherules in the lung tissue. (e) Left lung: innumerable diffuse tan nodules show miliary spread in the upper and lower lung lobes with predominance in the upper lobes in the lungs. The lungs were grossly boggy and congested; the left lung weighed 1250 grams (mean 400 grams). (f) Right lung: innumerable diffuse tan nodules showing miliary spread in the upper and lower lung lobes with predominance in the upper lobes in the lungs. The lungs were grossly boggy and congested; the right lung weighed 1400 grams (mean 450 grams). (g) Lymph nodes are diffusely enlarged and display a homogenous cut white/tan surface. (h) Axial plane view of the CT scan of the lungs showing military pattern of distribution of innumerable centrilobular and paraseptal pulmonary nodules in a random/miliary pattern of disease. (i) Axial plane of the CT scan of the abdomen and pelvis showing multiple enlarged lymph nodes along the periaortic distribution, right external iliac, and internal iliac chains.

His chest film on admission showed a diffuse micronodular pattern which triggered testing for COVID-19 which turned out to be negative. CT scan of the chest without contrast revealed extensive diffuse bilateral micronodules in a miliary pattern, mediastinal and axillary lymphadenopathy with pericardial thickening, while the CT scan of abdomen and pelvis revealed enlarged lymph nodes in the para-aortic, gastrohepatic, inguinal, external, and internal iliac chains (Figures 1(h) and 1(i)). While the patient was seen by many physicians to rule out lymphoma and tuberculosis, as well as to manage his respiratory failure, acute renal failure, and septic shock, the infectious disease physician suspected coccidioidomycosis and began a work up for the patient with serologic testing.

Since he was in shock, blood and urine cultures were sent and he was treated with IV isotonic fluid resuscitation with empiric intravenous vancomycin and cefepime along with intravenous methylprednisolone 60 mg every 8 hours and admitted to the intensive unit. Further testing showed that he was positive for HIV-1 with viral load 309,000 and CD4 count 38. We did not begin antifungal medications at the outset because we did not have a diagnosis of HIV at the time. Upon a diagnosis of HIV in the hospital, we expanded our differentials to miliary tuberculosis, histoplasmosis, and coccidioidomycosis. Histoplasmosis antigen, acid fast bacilli (AFB), and Cryptococcus antibody tests did not yield a positive result; however, sputum culture was positive for *Candida albicans*. Despite aggressive treatment with vasopressors, mechanical ventilation, and continuous renal replacement therapy, the patient died on hospital day 3. The family agreed to an autopsy which showed diffuse coccidioidomycosis with negative AFB stain and no evidence of malignancy. The cause of death was acute respiratory distress syndrome (ARDS) secondary to disseminated coccidioidomycosis secondary to HIV/AIDS infection.

### 2.1. Autopsy Findings

At autopsy, there were bilateral pleural lung effusions (left: 300ml, right: 400ml), congestion of the lungs with areas of consolidation, and extensive intra-alveolar edema. There were innumerable diffuse tan nodules showing miliary spread in the upper and lower lung lobes with predominance in the upper lobes in the lungs bilaterally (see Figures 1(e) and 1(f). These nodules microscopically consisted of granulomatous inflammation. The lung alveolar spaces were filled with proteinaceous material and Coccidioides fungal microorganisms. Coccidioides fungal microorganisms have spherules that may be empty or filled with endospores. Coccidioides fungal microorganisms are accentuated with Grocott-Gomori's methenamine silver stain (GMS) (see Figures 1(c) and 1(d)).The decedent grossly had extensive cervical, supraclavicular, inguinal, and axillary lymphadenopathy. The lymph nodes grossly showed a cut tan surface (see Figure 1(g)). Microscopically, normal lymph node architecture was replaced with caseating granulomas with Coccidioides fungal microorganisms that are accentuated with Grocott-Gomori's methenamine silver stain (GMS) (See Figures 1(a) and 1(b)). It should be noted that acid fast bacilli (AFB) stain was performed and was negative for acid fast microorganisms on both lung and lymph node sections.

### 2.2. Autopsy Findings and Figures

Autopsy Findings and Figures are presented in Figure 1.

## 3. Discussion

Coccidioidomycosis was first isolated in Buenos Aires, Argentina, by Alejandro Posadas in 1892; however, in the US, it was first documented in California. It is known to be endemic in California, Southern Arizona and New Mexico, Utah, Nevada, and Texas. The estimated incidence is greater than 150, 000 every year. It has been suggested that these numbers may not provide a true picture because health workers are not actively on the lookout for this disease on their list of differential diagnoses. Infections are usually self-limited. 60% of patients are asymptomatic or have mild respiratory illnesses [6, 8, 14].

According to medical literature, about 1% of sufferers will have their disease complicated and eventually develop disseminated coccidioidomycosis regardless of their immune status. Patients with the disseminated form have infections outside the lungs. Documented risk factors for this disease include HIV and AIDS, pregnancy, and immunosuppression. It is also known to be common in Africans and Filipino men and genetic and hormonal factors have been implicated [1, 8, 11]. People who work in occupations for which the soil is involved and disturbed or areas that are very dusty are at very high risk. Furthermore, workers exposed to high dust jobs such as farmers, construction personnel, and firefighters are also at risk [1, 11, 12]. Our patient worked for several years at a ceramic and tile factory which is not a documented occupational risk factor. While there should be a high index of suspicion in patients with fever, shortness of breath, lymphadenopathy, and pneumonia who reside in high risk areas; some patients present in extremis and deteriorate rapidly just like our patient did. Coccidioidomycosis can be misdiagnosed especially because of the COVID-19 pandemic and due to its similar presentation with tuberculosis or lung malignancies. In very severe cases, patients have had this disease invade the bones, spinal cord, brain, suprarenal gland, pancreas, mediastinum, skin, pelvic region, pericardium, and colon [1, 2, 8].

The reason for severe fatality in this disease is because the fungus causing this disease exhibits dimorphism, the ability to change the shape of their cells based on the temperature which enables their ability to be transformed. This transformation enables them morph into a spore-filled spherule potentially enabling invasion which pushes the immune system to defend the patient. For patients who recover, recovery can take several weeks to several months in healthy individuals. Other patients have this disease for the rest of their lives and have to be on lifelong antifungal medications. Significant mortality occurs with disseminated forms of this disease [3, 6, 15, 16].

Our patient presented in multiorgan failure and septic shock and these are unusual and infrequent presentations of coccidioidomycosis. Many studies have reported 100% mortality rate for this disease when patients present in shock [4]. Previous cases have documented similar presentations; however, the patient had a posterior mediastinal mass in which the patient developed respiratory failure, acute respiratory distress, and shock and died in the second week of presentation at the hospital [1].

Clinical clues that suggest coccidioidomycosis in our patient include the following: respiratory symptoms, significant generalized lymphadenopathy, eosinophilia of 11%, and absolute eosinophilia of 1260 (normal range is no more than 6%, count of no more than 350 in most laboratories) [3]. The patient resided in El Paso, Texas, an area affected by this condition but not top on the list for endemic areas as compared to California or Arizona. Because there are areas where coccidioidomycosis is not commonly seen, this disease may not be a top differential when patients present at the emergency department [5]. This disease is known to imitate many diseases, thus making diagnosis at the outset difficult [6, 17].

Per the Infectious Diseases Society of America (IDSA) 2016 clinical practice guidelines for HIV patients with a CD4^+^ T-lymphocyte count <250 cells/mL with coccidioidomycosis, the treatment recommendation is antifungal therapy until the count is above 250 cells/mL whether the patient is immunocompetent or not. The choice of antifungal therapy (fluconazole, itraconazole, or amphotericin B) does not differ for the immunocompromised patient [9, 10]. Amphotericin B however is preferable in severe disease. While there is a risk of immune reconstitution inflammatory syndrome (IRIS) in coccidioidomycosis management, antifungal medication should be initiated in the patient. Irrespective of HIV status, fluconazole or itraconazole is the treatment of choice for nonmeningeal extrathoracic disseminated disease [9]. Medical literature has shown that itraconazole is more efficacious than fluconazole for the treatment of disseminated disease. Amphotericin B is used for azole failure and/for patients with severe disease. In a randomized controlled trial, after 8 months of treatment, itraconazole group patients were reported to have had more response (63% versus 47%) and less relapse (18% versus 28%) when compared to fluconazole group patients after 12 months of treatment [18]. Amphotericin B is used for azole failure and/or patients with severe disease such as lesion in the spine or a peritracheal abscess with airway compromise. Within coccidioidomycosis endemic regions, patients should receive yearly serologic screening and chest radiography for the disease [9]. Unfortunately, even with treatment, many patients still die.

Regardless of presentation, it is imperative to consider that this disease can have multiple presentations and a careful history including travel history and immune compromise may assist in the diagnosis of this disease [7].

## 4. Conclusion

The symptoms of coccidioidomycosis overlap several conditions, affect multiple organs, and therefore imitate various other ailments. Significant effort is required to discern the appropriate therapeutic intervention to achieve resolution. Recognition of extrapulmonary manifestation of this disease is important. This patient had vague symptoms, eosinophilia and lymphadenopathy, so we considered infectious diseases, neoplastic disorders and COVID-19.

According to the CDC, even though *Coccidioides* is present in western Texas, coccidioidomycosis is not a reportable disease, except in the city of El Paso. Since there is no requirement for this disease to be reported statewide, this may be a reason why physicians are not on the aggressive lookout for it. There have been a few initiatives at the health departments at the county level for surveillance for this disease; however, they have reported only 12 cases in 2015, or 1.4 cases per 100,000 population in El Paso county [19, 20]. The authors suggest that this disease becomes a reportable disease at the national level even though it has only been reported in small numbers in western Texas.

## Data Availability

The image (autopsy findings and figures) data used to support the findings of this study are included within the article.
